# Symmetric and Asymmetric Magnetic Tunnel Junctions with Embedded Nanoparticles: Effects of Size Distribution and Temperature on Tunneling Magnetoresistance and Spin Transfer Torque

**DOI:** 10.1038/s41598-017-08354-7

**Published:** 2017-08-21

**Authors:** Arthur Useinov, Hsiu-Hau Lin, Chih-Huang Lai

**Affiliations:** 1 Department of Physics, National Tsing Hua University, Hsinchu, Taiwan; 2 Department of Materials Science and Engineering, National Tsing Hua University, Hsinchu, Taiwan; 3 Institute of Physics, Kazan Federal University, Kazan, Russian Federation

## Abstract

The problem of the ballistic electron tunneling is considered in magnetic tunnel junction with embedded non-magnetic nanoparticles (NP-MTJ), which creates additional conducting middle layer. The strong temperature impact was found in the system with averaged NP diameter *d*_av_ < 1.8 nm. Temperature simulation is consistent with experimental observations showing the transition between dip and classical dome-like tunneling magnetoresistance (TMR) voltage behaviors. The low temperature approach also predicts step-like TMR and quantized in-plane spin transfer torque (STT) effects. The robust asymmetric STT respond is found due to voltage sign inversion in NP-MTJs with barrier asymmetry. Furthermore, it is shown how size distribution of NPs as well as quantization rules modify the spin-current filtering properties of the nanoparticles in ballistic regime. Different quantization rules for the transverse component of the wave vector are considered to overpass the dimensional threshold (*d*_av_ ≈ 1.8 nm) between quantum well and bulk-assisted states of the middle layer.

## Introduction

Magnetic tunnel junctions (MTJs) are well-known electronic devices, which can be applied as a magnetic field sensors^1, 2^, read heads of hard drives^3^, electronic compass, automotive sensors^4^, etc. In particular, scanning MTJ microscope was constructed for a high-resolution imaging of remanent magnetization field^5^. Furthermore, magnetic random access memory (MRAM) prototype operated by spin transfer torque (STT) was made currently on the basis of single barrier magnetic tunnel junction (SMTJ)^6, 7^. The tunneling barrier should be as thin as possible to achieve a low resistance area product (RA), the device should keep the satisfied thermal stability factor with an acceptable noise to signal ratio as well as tunneling magnetoresistance (TMR) magnitude around 100%. The reduction of writing current density is still actual problem, from this point of view, the studies of the complex barrier is an important issue which assume to find any benefits to reduce writing current density. In turn, it may reduce the energy consumption. However, the barrier thickness decreasing less than 1.0 nm may results in significant drop of the breakdown voltage. The writing endurance also rapidly decreases due to low tolerance to the defects. Therefore, interlayer exchange coupling between ferromagnetic layers (FMLs) becomes larger in SMTJ, whereas the barrier height decreases. Thermal stability factor and critical switching current are affected by the interlayer interaction and spin transfer torque^7, 8^.

Present research is oriented to study a composite barrier-type structure such as magnetic tunnel junctions with embedded non-magnetic nanoparticles (NP-MTJ) at finite temperatures. These junctions are comparable with SMTJs in terms of high thermal stability factor, TMR and low RA values. The tunneling current and TMR amplitude significantly depend on the conditions related to size distribution of NPs, quantization, dispersion relations and temperature. The main goal of this work is to highlight an important conditions for the dimension and voltage induced thresholds as well as demonstrate TMR and STT behaviors due to presence of NPs. All NP-MTJ characteristics are similar to those in double barrier magnetic tunnel junctions (DMTJs), since the basic tunneling phenomena is the same. Double barrier MTJs are promising structures for MRAM applications due to higher thermal stability factor, low noise and less critical current density for the switching in comparison to SMTJs^9–15^.

The present work is based on the assumption of the ballistic transport in NP-MTJs, which were considered as a chain of the double barrier tunneling cells (TCs) connected in parallel. Experimental works by Yang *et al*.^14^ and Ciudad *et al*.^15^, where TMR anomalies were found, were considered as a data sources for the comparison with our results. Yang *et al*. considered NP-MTJ with in-plane magnetic anisotropy on the basis of SiO_2_/Ta(10)/Ir_22_Mg_78_(25)/Co_70_Fe_30_(3.5)/Mg(0.8)/MgO(2.5)/Co_70_Fe_30_(*t*)/Mg(0.8)/MgO(2.5)/Co_70_Fe_30_(7)/Ir_22_Mg_78_(15)/Ta(10) structure (thicknesses in nm), which contains encapsulated Co_70_Fe_30_ nanoparticles inside MgO layer. This structure was characterized by the middle layer thickness *t*, the values from 0.25 nm to 0.75 nm are corresponded to the average NP diameters *d* = 1.5 ± 0.4 nm to *d* = 3.2 ± 0.7 nm, respectively. Parameter *t* is nominal and assigned to ideal conditions of deposition of the homogeneous middle layer, while formation of NPs and their size distribution are result of the clusterization. The distribution of the embedded NPs and its formation might be controlled by deposition rates, materials and annealing conditions. The simplest way to find the NP size distribution is to make the tunnel electron microscope (TEM) images of the front interface and cross-section of the middle layer in the sample.

Yang *et al*.^14^ and Ciudad *et al*.^15^ suggested to explain the observed anomalous TMR voltage behaviors applying the models of consecutive tunneling, Kondo-assisted co-tunneling as well as Coulomb blockade (CB) effects, but neglecting the direct (ballistic) tunneling. According the tunneling model shown by Ciudad *et al*. the tunneling conductance can be represented as the summation of the three terms: *σ* = *aσ*_*D*_ + *b*(*σ*_*E*_ + *σ*_*K*_), where *a* + *b* = 1, *σ*_*K*_ is the conductance through the NPs due to Kondo effect; *σ*_*D*_ and *σ*_*E*_ are the direct tunneling and elastic conductance through the NPs without spin flips. The contribution for *a* and *b* depends on the fractional populations of clusters and temperature. The conditions also relate with barrier thickness: it assumes *a* > *b* for the thin layers. The critical thickness for *a* > *b* is not defined, but assumed that *t* → 0, *a* → 1, while for *t* ~ 3 nm, *a* → 0 and thus *b* → 1, and, in turn, second term in *σ* becomes dominant. It also suggested *σ*_*K*_ ≫ *σ*_*E*_ at *t* ~ 3 nm, but for further *t* increasing it results to *σ*_*E*_ ~ *σ*_*K*_ and *σ*_*E*_ ≫ *σ*_*K*_. The competition between *σ*_*K*_ and *σ*_*E*_ reflects TMR dependence and the changes of its amplitudes from reduced to enhanced one at small temperatures and applied voltages. However, nevertheless on the suggested approach with *t* ≥ 3 nm, Ciudad considered the samples *t* < 1.2 nm, yet assuming *a* → 0. Moreover, there is no critical size estimations to assume *b* → 1 for the considered cases at *t* < 1.2 nm.

It is worth to notice, that formation of the homogeneous layer due to deposition or epitaxial growth of the middle layer can be realized at *t* ~ 1.6–2.0 nm, avoiding the case of the dominated and separated clusters with *d*_av_ ~ 10 nm (*t* ~ 3 nm), where CB might be important. According to the experimental work^16^, it was concluded that low capacitance in NP-MTJs rules out any charging effects. In addition, it was observed unexpected anomalous TMR enhancement in NP-MTJ with Fe nanoparticles at *t* < 1.0 nm, that probably relate to quantized conductance: an attempt to apply CB approach was not successful too^10^. Moreover, according fist principal calculations, which are shown in supplementary materials of the present work, the density of the states (DOS) for the small NP (*d* = 1.14 nm) is large enough to be over the CB effects. Another arguments which support the direct tunneling model are also collected in supplementary materials.

In our previous works^17–19^ we showed that regime of the quantized conductance due to direct double barrier tunneling (*a* = 1) is a main reason of anomalous TMR behaviors, confirming the lack of CB effects for the small NPs, *d* < 3.5 nm, in these systems. Our basic tunneling approach has similar origin with the theory of giant magnetoresistance in point-like contacts^20^ reproducing the limit of the point-like contact when NP touches top and bottom FMLs. However many aspects of the problem related to variation of barrier asymmetry, size distribution, quantization of the states and temperature factors were not completely studied. In turn, the present work shows the contrast between different quantization rules and gradual transitions between NP size distributions, opening a hidden thresholds and related TMR behaviors. Furthermore, simulation of asymmetric NP-MTJ, in which top and bottom barrier widths *L*_1(2)_ are not equal to each other, predicts a large voltage sign asymmetry for STT behavior.

### Theoretical model

**Figure 1:**
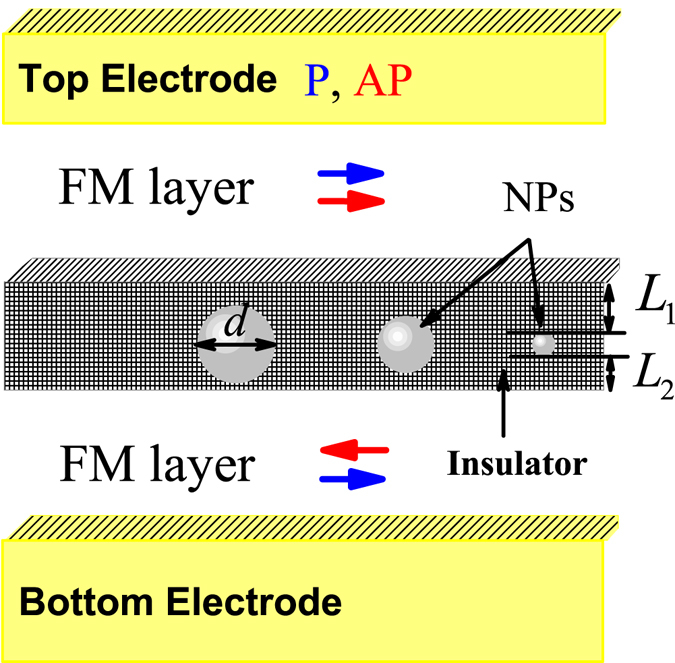
Schematic model of the NP-MTJ. Blue and red arrows show parallel (P) and anti-parallel (AP) magnetic configurations, respectively.

Magnetic tunnel junction with embedded non-magnetic nanoparticles was simulated as one hundred TCs connected in parallel (*N*_tot_ = 100). A few TCs are shown schematically in Fig. 1, each TC contains one NP. Ten fractions *f* = 1.10 were considered with diameter *d* ^(*f*)^, and ${\sum }_{f}{i}_{f}=100$, where *i* is a number of NPs. The *fraction* is defined as a set of TCs with the same size of NPs. The ballistic tunneling was considered as a basic transport approach, and the current for each TC was defined as follows^18, 19^:

1 $$\begin{array}{c}{I}_{s,f}=\frac{{e}^{2}}{h}\frac{{k}_{F,s}^{2}{[{d}^{(f)}]}^{2}\,V}{8}\\ \,\quad \,\times {\int }_{{X}_{1}}^{{X}_{2}}dx{\int }_{0}^{{\theta }_{min}}sin({\theta }_{s})cos({\theta }_{s}){D}_{s}({\theta }_{s},{k}_{n}^{(f)}+x)d{\theta }_{s},\end{array}$$

where *V*, *s* and *θ*_min_ - applied voltage, spin index, and the limit of incident angle *θ*_*s*_, which arises from conservation of the longitudinal projections of the *k*-vectors to the interfaces; ${X}_{1(2)}=\mp \sqrt{2{m}_{{\rm{eff}}}\,{k}_{{\rm{B}}}T/{\hslash }^{2}}$, where *m*_eff_, *k*_B_ and *T* are effective electron mass, Boltzmann constant and temperature, respectively. Equation 1 consists integrals over the top incident electron trajectory angle *θ*_*s*_ and temperature-related broadening; *k*_*F*_,_*s*_ is defined as a Fermi wavenumber of the top FML for positive voltage. The derived (1) is more accurate than Wentzel - Kramers - Brillouin (WKB) method^21^, since the transmission coefficient of the double barrier system *D*_*s*_ was obtained as exact analytical solution^22^ for the double barrier system, that take in consideration the quantized levels in the middle conducting layer. Transmission is a function of *k*_*F*_,_*s*_, *θ*_*s*_ and transverse wavenumber through the nanoparticle, *k*_*n*_.

**Figure 2:**
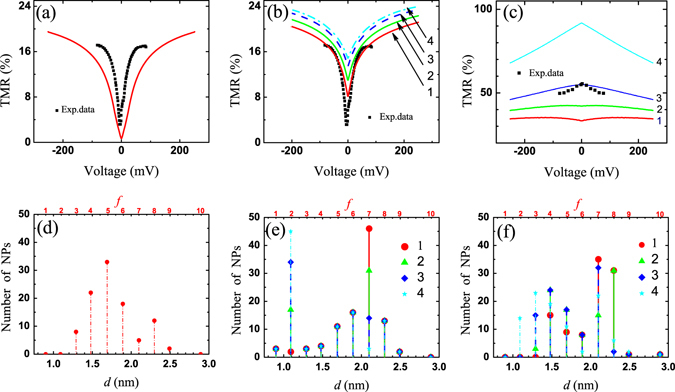
TMR-*V* dependencies with related size distributions. (**a**) The TMR-*V* with reduced TMR_0_ is shown for $n=1$, ${\rm{\Phi }}=0.1{{\rm{nm}}}^{-{\rm{1}}}$ in comparison to exp. data^14^ at *t* = 0.45 nm with size distribution similar to panel (**d**). (**b**) TMR-*V* curves variations, *n* = 1 and ${\rm{\Phi }}=0$. (**c**) TMR-*V* curves at n = 2 and ${\rm{\Phi }}=0$ for different size distributions. (**d**) Size distribution corresponds to the theoretical solid curve in panel (**a**). (**e**) and (**f**) panels show distributions for 1–4 curves of the panel (**b**) and (**c**), respectively. Black squares in panel (**c**) are extracted exp. data in Fig. 4 (ref. 14) at *t* = 1.79 nm. All presented data were derived at *T* = 2.5 K.

**Figure 3:**
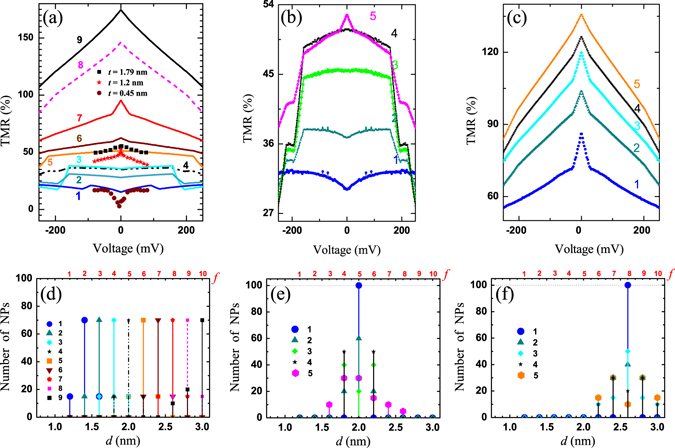
(**a**) TMR-*V* curves in case of low degree of quantization. Colored symbols (circles, stars and squares) are exp. data^14^ for *t* = 0.45 nm, *t* = 1.2 nm and *t* = 1.79 nm, respectively. (**b**) and (**c**) show the TMR-*V* curves for the system with average NPs sizes *d*_av_ = 2.0 nm and *d*_av_ = 2.6 nm, respectively. Detailed description of the curves is given in the text. (**d**), (**e**) and (**f**) represent the size distributions of TMR curves depicted in (**a**), (**b**) and (**c**) panels, respectively.

**Figure 4:**
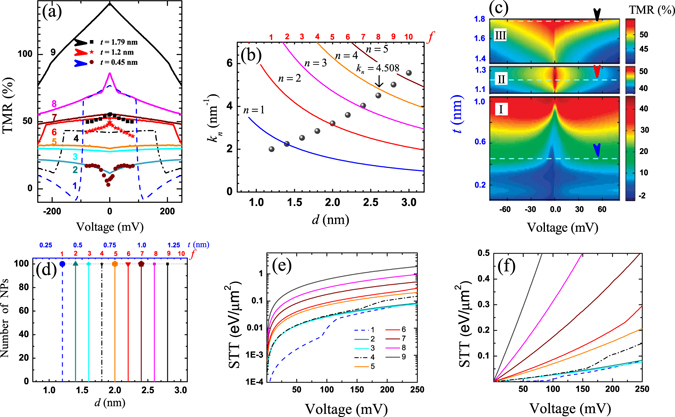
(**a**) TMR-*V* in case of low degree of quantization. Brown circles, red stars and black squares are exp. data. (**b**) The dark beads show initial distribution over *k*_*n*_. Color solid curves show ideal QW solution ${k}_{n}^{(f)}=\pi n/{d}^{(f)}$ for *n* = 1 to *n* = 5, respectively. (**c**) Experimental data^14^, *T* = 2.5 K. (**d**) The distribution of the NPs number over size. (**e**) and (**f**) show voltage dependences of the logarithmic and linear scales of the in-plane STT components, which correspond to TMR-*V* curves of the panel (**a**), respectively.

**Figure 5:**
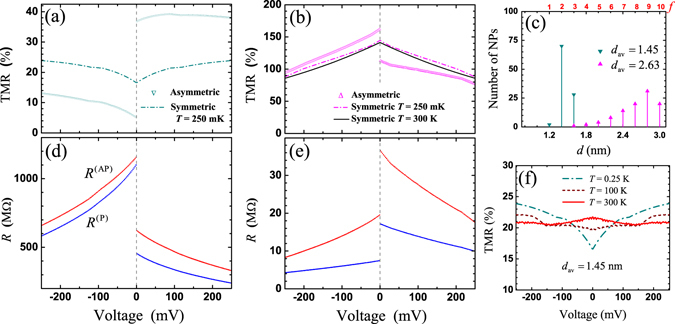
TMR voltage dependencies for symmetric ${L}_{\mathrm{1(2)}}=1.0$ nm and asymmetric cases. Asymmetric parameters ${L}_{1}=1.0$ nm, ${L}_{2}=0.9$ nm for (**a**) and ${L}_{1}=0.9$ nm, ${L}_{2}=1.0$ nm for (**b**) correspond to ${d}_{{\rm{av}}}=1.45$ nm and ${d}_{{\rm{av}}}=2.63$ nm, respectively. The related asymmetric resistance behaviors of the TMR curves depicted in (**a**) and (**b**) are shown in (**d**) and (**e**), respectively. (**c**) Size distributions. (**f**) Temperature dependence of the symmetric TMR behavior at ${d}_{{\rm{av}}}=1.45$ nm.

**Figure 6:**
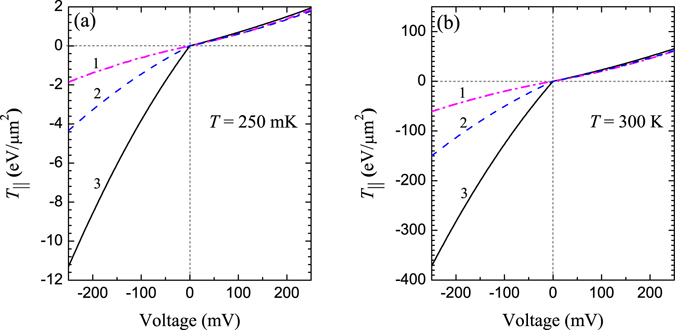
Symmetric and asymmetric STT voltage dependence at $T=250$ mK (**a**) and $T=300$ K (**b**), respectively. The bottom barrier width is fixed, ${L}_{2}=1.0$ nm. Curves 1–3 correspond to symmetric ${L}_{1}=1.0$ nm and asymmetric ${L}_{2}=0.9$ nm, ${L}_{1}=0.8$ nm cases for ${d}_{{\rm{av}}}=2.63$ nm, respectively.

It is important to notice that ballistic conductance shows latent temperature dependence. Temperature related impact, accounted by integration over *x*, reflects a conduction band broadening, since electron energy ${E}_{n}={\hslash }^{2}{k}_{n}^{2}\mathrm{/2}m$ might be comparable to *k*_*B*_ *T*. The wavenumber values for majority and minority electron bands were assumed as initial FML parameters at zero voltage ${V}_{0}=\pm {10}^{-4}\,{\rm{V}}$ for all considered NP-MTJs as ${k}_{F,\uparrow }=10.9{{\rm{nm}}}^{-1}$ and ${k}_{F,\downarrow }\mathrm{=4.21}\,{{\rm{nm}}}^{-1}$, respectively. TMR at this voltage was determined as TMR_0_. The absolute zero voltage point, *V* = 0.0 V, was kept as unsolved one for all theoretical simulations illustrated in Figs 2–6.

To consider the positive voltage impact and spin filtering effect of NPs, electron wavenumbers in NPs and bottom FML were modified as follows:

2 $$\begin{array}{c}{k}_{n}^{(f)}(V)={[{({k}_{n}^{(f)})}^{2}+c\cdot ({m}_{{\rm{eff}}}/{m}_{0})\cdot {V}_{1}]}^{\mathrm{1/2}},\\ {k}_{F,s}(V)={({k}_{F,s}^{2}+c\cdot {V}_{2})}^{\mathrm{1/2}},\end{array}$$

where ${V}_{\mathrm{1(2)}}=V\cdot {\varepsilon }_{\mathrm{2(1)}}{L}_{\mathrm{1(2)}}/({\varepsilon }_{2}{L}_{1}+{\varepsilon }_{1}{L}_{2})$ is voltage drop in the first (second) barrier with applied total voltage *V*; ${\varepsilon }_{\mathrm{1(2)}}$ and *L*_1(2)_ - dielectric constants and barriers width, $c\,=\,2{m}_{0}e/{\hslash }^{2}\cdot {10}^{-18}\approx 26.25$ ${{\rm{nm}}}^{-{\rm{2}}}\,{{\rm{eV}}}^{-{\rm{1}}}$ is the dimensional factor, and *m*_0_ is free electron mass. The symmetry of the system was used for the model simulations at negative voltages, ${V}_{\mathrm{1(2)}}\to {V}_{\mathrm{2(1)}}$. The transverse wavenumber ${k}_{n}^{(f)}$ in NP is the key parameter, since the result of integration (1) is very sensitive to its value. The related choice of initial ${k}_{n}^{(f)}$ was varied from 1.0 nm^−1^ to ~6.0 nm^−1^. The quantum well solution, which was used as initial approach of ${k}_{n}^{(f)}$ distribution over *d*, is presented below:

3 $${k}_{n}^{(f)}=\pi n/{d}^{(f)}-{{\rm{\Phi }}}^{(f)},$$

where correction term ${{\rm{\Phi }}}^{(f)}$ places ${k}_{n}^{(f)}$ aside from ideal quantum well (QW) solution corresponding to the real systems. Index *n* is obtained as an integer number corresponding to QW discrete levels. Equation (3) is simplified quantization rule, $2\,{k}_{n}d+{{\rm{\Phi }}}_{B}+{{\rm{\Phi }}}_{T}=2\pi n$, and term ${{\rm{\Phi }}}_{B(T)}$ is the phase change of the electron wave function on interfaces^11, 12^. However, tunneling behavior at $d > 1.8{\rm{nm}}$ can be different due to increased NP volume, and then NP reveals more bulk-assisted properties, whereas degree of quantization is vanished. Quantization rule ${k}_{n}^{(f)}(d)$ can be simulated as slight parabolic dependence, where index *n* becomes nominal. The simulations with QW quantization are considered only at low temperature approximation (*T* = 0.25–2.5 K) in the next section (in the subsection Approach of quantization over QW states). Parabolic quantization rule was applied in another subsections B to F for low and high temperatures. All main results depicted in Figs 2, 3a, 4a and 5 were found according to TMR definition as follows: ${\rm{TMR}}=({I}^{{\rm{P}}}-{I}^{{\rm{AP}}})/{I}^{{\rm{AP}}}\times \mathrm{100 \% }$, where ${I}^{{\rm{P}}({\rm{AP}})}=\sum {i}_{f}\cdot ({I}_{\uparrow ,f}^{{\rm{P}}({\rm{AP}})}+{I}_{\downarrow ,f}^{{\rm{P}}({\rm{AP}})})$, and each term ${I}_{s,f}^{{\rm{P}}({\rm{AP}})}$ was calculated by (1); the resistance of the system: ${R}^{P(\mathrm{AP})}=V/{I}^{P(\mathrm{AP})}$. The tunneling current, which passes through insulator between NPs, was considered as negligible impact. The barriers thicknesses in symmetric NP-MTJs were fixed as ${L}_{\mathrm{1(2)}}=1.0\,{\rm{nm}}$, dielectric constants ${\varepsilon }_{\mathrm{1(2)}}=10.0$ and ${m}_{{\rm{eff}}}=0.8{m}_{0}$ for NPs, ${m}_{{\rm{eff}}}=0.4{m}_{0}$ for barriers, and ${m}_{{\rm{eff}}}={m}_{0}$ for FMLs.

Among different insulating materials MgO barrier is more complex one rather than others, e.g. Al_2_O_3_, due to complicated band structure. Single crystal MgO induces an enhancement of the spin polarization *P* of the tunneling current, filtering mostly ${k}_{F,\downarrow }$ and related wave functions^23, 24^. In present work, the band structure or complex *k*-vector behavior for the barriers were not considered, but it is assumed that ${k}_{F,\uparrow }$ and ${k}_{F,\downarrow }$ values are effective, that take into account the influence of the MgO spin-filtering properties. In particular, ${k}_{F,\downarrow }$ value is reduced in relation to real one in FM layers. Experimental and theoretical estimations for *k*-values is accessed in refs 25 and 26, respectively. The *P* = 0.443 was obtained for our initial parameters according definition: $P=({k}_{F,\uparrow }-{k}_{F,\downarrow })/({k}_{F,\uparrow }+{k}_{F,\downarrow })$. In case of Al_2_O_3_ based MTJ^15^ additional spin-filtering effect is absent in comparison to MgO based junction that assume a valuable *P* decreasing (e.g. *P* = 0.2–0.3 at ${k}_{F,\downarrow }=$ 5.5–7.0 nm^−1^). The low TMR amplitude with maximum around 10–25% is well observed in ref. 15 that also confirmed by our calculations. Nevertheless, all voltage and temperature behaviors as well as related quantum effects are similar with MgO based junctions.

One of important dynamic MTJ properties of the magnetic distortion is STT effect^7, 27, 28^. Spin transfer torque is determined by vector which consists of parallel *T*_||_ and perpendicular *T*_⊥_ components in relation to interfaces. The parallel component *T*_||_ vanishes at zero voltage, while at finite voltage it lies in the plane of FML interfaces triggering additional magnetization fluctuation. The value of *T*_⊥_ is determined by interlayer exchange interaction, which isn’t vanished at zero voltage, ${T}_{\perp }\ne 0$. Only *T*_||_ component will be considered below, since we assumed in our case ${T}_{||}\gg {T}_{\perp }$. Parallel STT component is calculated as difference between spin current densities ${\Im }^{{\rm{P}}(\mathrm{AP})}$, refs 27, and 28:

4 $${T}_{||}=\frac{\sin (\gamma )}{2}({\Im }^{{\rm{P}}}-{\Im }^{{\rm{AP}}}),$$

where ${\Im }^{{\rm{P}}(\mathrm{AP})}=\hslash ({J}_{\uparrow }^{{\rm{P}}(\mathrm{AP})}-{J}_{\downarrow }^{{\rm{P}}(\mathrm{AP})})\mathrm{/2}e$, and *γ* is angle between FMLs magnetizations; ${J}_{s}^{{\rm{P}}(\mathrm{AP})}={I}_{s}^{{\rm{P}}(\mathrm{AP})}/{S}_{{\rm{\phi }}}$ is the charge current density, and, finally, ${S}_{{\rm{\phi }}}=\frac{\pi }{4}\sum _{f=\mathrm{1..10}}{i}_{f}{[{d}^{(f)}]}^{2}$ is the total active cross-section area of the NP-MTJ.

## Results

### Approach of quantization over QW states

Figure 2a,b and c show the experimental data fitting which was made according to the presented theoretical approach (1) and distribution (3), e.g. Figure 2a correspond to *n* = 1 and ${\rm{\Phi }}=0.1\,{{\rm{nm}}}^{-{\rm{1}}}$. The size distribution, Fig. 2d, is close to experimental data, where dip-like TMR behavior was observed only at low temperatures, *T* ≈ 2.5 K ref. 14. Figure 2b show series of the suppressed TMR_0_ behaviors, derived at ${k}_{n}\approx 1.26$–$3.14\,{{\rm{nm}}}^{-1}$, *n* = 1 and ${{\rm{\Phi }}}^{(f)}\mathrm{=0}$. Its NP size distribution, Fig. 2e, demonstrates how NPs numbers *i*_*f* = _2 and *i*_*f* = 7_ is changed in opposite way: TCs with *d* = 1.09 nm increases from 3 to 45 pieces in contrast to the same amount decreasing for *d* = 2.098 nm. The lowest TMR_0_ (curve 1) are created under the fraction with lowest ${k}_{n}^{(f)}$ and largest *d* (${i}_{f=7}\gg {i}_{f=2}$). Thus, the suppressed TMR_0_ is a result of low *k*_*n*_ with quantization by the order of first or second QW levels. We also found that tunneling cells with the largest NPs by size provide the most significant contribution to the total ballistic current.

Tunneling magnetoresistance as a function of the applied voltage is strongly modified at *n* = 2, Fig. 2c. The gradual TMR variations show initially suppressed TMR at low voltages (curve 1) and then dome-like behavior (curve 4) depending on the size distribution. Figure 2f represents the competition between relatively small (*d* < 1.7 nm) and middle (1.7 nm–2.5 nm) size NP’s fractions. All initial *k*_*n*_ are different for each fraction according to (3). The formation of the lowest TMR_0_, with dip-like behavior, follows to the fraction with lowest ${k}_{n}^{(f)}$ (curve 1) that is similar to the case *n* = 1. The lowest ${k}_{n}^{(f)}$ values corresponds to NPs distribution with greatest averaged NP diameter: ${d}_{{\rm{av}}}=\frac{1}{{N}_{{\rm{tot}}}}\sum {i}_{f}{d}^{(f)}$. The calculated TMR curves satisfactorily reproduces experimental data at this case [e.g. Fig. 4 and Fig. S6 in ref. 14], however, TMR behavior (in relation to parameter *t*) is opposite to experimental observations for *d*_av_ > 1.8 nm. Thus, it might be more rational explanation when *k*_*n*_ also increases with *d*_av_. It is assumed, that there is a size threshold at *d*_av_ ≈ 1.8 nm (*t* ≈ 0.8 nm) between quasi-1D and bulk-assisted states in the middle conducting layer, and thus, quantization rule (3) have to be changed.

### Approach of parabolic quantization rule

To reproduce an experimental data more precisely as a matter of correlation between growing middle thickness *t* and TMR respond, an initial *k*_*n*_ distribution with low quantization degree was considered as a slight parabolic dependence for 1.1 nm < *d* < 3.5 nm (dark beads in Fig. 4b). Nevertheless, this distribution can be also treated as QW quantization (solid lines in Fig. 4b) corrected with Φ. Complete form of TMR behavior with maximum number of NPs for each fraction is shown in Fig. 4a, while Fig. 3a predicts TMR as a result of the contributions from the nearest fractions. The circles, stars and squares symbols in Figs 3a and 4a show extracted experimental data of numerated regions of the Fig. 4c. According to results, the curves 1–4 in Fig. 3a and curves 2, 3, 5 in Fig. 4a fit the region I of experimental output, Fig. 4c. Curves 6 and 7 in Fig. 3a are similar to curve 8 in Fig. 4a, which correspond to the region II. Finally, curves 8, 9 in Fig. 3a and curve 9 in Fig. 4a are assigned to the region III. Similar experimental behaviors for all these regions can be found in experimental work^15^ too. As a result, presented theoretical model is able to reproduce experimental TMR behaviors with respect to NPs size and *k*_*n*_ distributions as well as predict step-like TMR and STT quantized behaviors derived at low temperature. The reduced experimental TMR values, in relation to theoretical one, might be related with uncounted spin-flip leakages as well as *γ* ≠ *π* for multi-domain structures.

The result of TMR variations due to different size distributions related to the same constant NP diameter (*d*_av_ = const) is presented in Fig. 3b (*d*_av_ = 2.0 nm) and Fig. 3c (*d*_av_ = 2.6 nm). The curve 1 in Fig. 3b shows suppressed TMR_0_ due to low *k*_*n*_ = 3.2 nm^−1^ value, which is similar to TMR behavior in region I, Fig. 4c. Furthermore, peak-like TMR_0_ enhancement is observed when the fraction with resonant parameters *d* = 2.6 nm and *k*_*n*_ = 4.08 nm^−1^ becomes a part of distribution, e.g. curves 6 and 7 in Fig. 3a and curve 5 in Fig. 3b. These parameters are close to the origin of the quantized conditions for *I* ^P(AP)^ in related voltage range. It is clearly shown how TMR_0_ increases when size distribution becomes less uniform in both Fig. 3b and c, the valuable impact is induced here from TCs with largest *k*-values.

As a result, the valuable TMR_0_ corresponds to the fractions with a largest NP diameters, since *k*_*n*_ gradually increases according related size distribution. Resistance area product decreases rapidly from a few MΩ·*μ*m^2^ at *d*_av_ < 1.1 nm to a few kΩ·*μ*m^2^ at *d*_av_ = 2.5 nm. TMR behavior at small voltage is consistent with experimental observations. Finally, we demonstrated that homogeneously (uniformly) deposited middle layer between barriers with largest thickness, which is still correspond to ballistic tunneling regime, is an important condition for the largest TMR and lowest RA values.

### Quantized TMR and STT behaviors

The threshold voltages, which result in step-like TMR behaviors and depicted in Fig. 3a, e.g. curves 1–4 at *V* ≈ 90 mV–200 mV and Fig. 3b with curves 2–5 at *V* ≈ 170 mV–$190$ mV, are predicted here. The voltage threshold occurs by cause of restricted NPs geometry and condition of the quantized conductance. The additionally opened conduction channels abruptly change the current relation between *I* ^P^ and *I* ^AP^ at the threshold. Moreover, the reason of the step-like TMR curve 1 in Fig. 4a, is assumed to be different and related with *k*_*n*_ threshold. Since *k*_*n*_ becomes too small, electron wave function shows deficiency of any quantum interference for *f* = 1, *d* = 1.2 nm at *V* ≈ 10^−2^–10^2^ mV. Conduction electrons are involved only in single barrier tunneling, since electron wavelength is large enough. The conditions of tunneling is drastically changed regarding *k*_*n*_ and *k*_*F*, s_ behaviors with applied voltage. The double barrier direct tunneling arises at voltage threshold |*V*| > 100 mV again, shifting TMR magnitude into the negative range. Similar TMR behavior is weakly highlighted in Fig. 4c, *t* < 0.2 nm, but single barrier tunneling voltage range is a few mV, and it is much smaller in comparison to theoretical curve 1, Fig. 4a.

The in-plane component of STT maximal amplitude was calculated at *γ* = *π*/2, Fig. 4e and f. Curves 1 and 4 correlate with related TMR curves (Fig. 4a) which have abrupt steps as a result of the mentioned thresholds. STT steps potentially can be used in spintronic devices for the voltage control of magnetic states. Moreover, STT has a strong dependence *via d* at finite *V*. For example, STT values are rapidly changed due to the growing NP size by one order from ~0.1 to ~1.0 eV *μ*m^−2^ at *V* = 250 mV, which correspond to the curves 1 to 9, respectively.

### Approach of asymmetric NP-MTJ

The model is also convenient to TMR simulations in asymmetric NP-MTJs, non-equal voltage drop is proportional to the barrier width. An example of TMR curves are shown in Fig. 5a and b for ${L}_{1}=1.0$ nm, ${L}_{2}=0.9$ (${d}_{{\rm{av}}}=1.45$ nm) and ${L}_{1}=0.9$ nm, ${L}_{2}=1.0$ (${d}_{{\rm{av}}}=2.63$ nm), respectively. The related TMR behaviors are characterized by asymmetric *R*-*V* curves which are shown in Fig. 5d and e, respectively. The resistance value *R* is derived for 100 TCs connected in parallel, where $R\propto {S}_{\varphi }^{-1}$. The problem R → 0 in asymmetric NP-MTJ is considered insupplementary material with presence of the additional intrinsic voltage bias on the interfacesThe negative and positive TMR-*V* curves reproduce TMR asymmetric behaviors which are highlighted for experimental data in Fig. 4c at $t\approx 0.5$ nm (region I) and $t\approx 1.6$ nm (region III), respectively. The quantization rule is kept as parabolic one, and size distribution is shown in Fig. 5c. Consideration of the randomly generated distributions over *L*_1_ and *d* in range of 0.9 nm $< \,{L}_{1} < 1.1$ nm (${L}_{2}=1.0$ nm) and 1.2 nm < *d* < 3.0 nm leads to the similar asymmetric TMR effects. The divergence in real structures is result of lattice defects in barriers and not-ideal shapes of NPs. The defect size might be even less than dimension of lattice constant, e.g. presented simulations shows how even one angstrom difference in barrier width gives sensitive impact.

### Temperature dependence

Figure 5f shows TMR evolution with temperature in symmetric NP-MTJ at ${d}_{{\rm{av}}}=1.45$ nm. The transition from TMR dip-like to a weak dome-like behaviors occurs due to temperature variation from 250 mK to 300 K, respectively. Thus, temperature dependence correctly reflects an experimental results, whereas TMR_0_ dip was observed only at low temperatures^14, 15^. The temperature dependence is strong for *f* = 1–3, since related *k*_*n*_ values in these fractions are relatively small. A weak temperature dependence is observed in another case ${d}_{{\rm{av}}}=2.63$ nm for *f* = 4–10, Fig. 5b. Moreover, if consider peak-like (e.g. curve 1 in Fig. 3c, curve 8 in Fig. 4a) and step-like TMR (e.g. curves 2–5, Fig. 3b) behaviors, the results will be strongly averaged by room temperature representing classical dome-like behaviors. It was also noticed that TMR peak-like behavior decreases with temperature, and it can be more stable with increasing temperature in comparison to TMR_0_ dip. For example, experimental data in ref. 15, which are presented in [Fig. 3c,e and f], also clearly show how width of TMR_0_ peak varies with temperature, it accompanies by slowly decreased amplitudes. In scope of our model, TMR maximum obtained for Al_2_O_3_ is much lower than for MgO barrier. The related amplitudes in 3% for dip-like (at $V=200$ mV) and TMR_0_ = 16.6% for dome-like TMR behaviors at *T* = 250 mK were found in assumption *P* = 0.27 (${k}_{F,\uparrow }=10.9{{\rm{nm}}}^{-1}$ and ${k}_{F,\downarrow }=6.21{{\rm{nm}}}^{-1}$) for ${d}_{{\rm{av}}}=1.45$ nm and ${d}_{{\rm{av}}}=2.63$ nm, respectively. Finally, TMR simulation at relative large ${k}_{n} > 5.0\,{{\rm{nm}}}^{-1}$ results in almost identical dome-like TMR curves at low as well as at room temperatures, which are similar to those obtained for symmetric junctions, Fig. 5b.

### Asymmetric STT behavior

Induced in-plane STT was found in the bottom FML for the different cases of barrier asymmetry at finite temperatures. In particular, curve 3 in Fig. 6 shows that difference of the negative and positive STT voltage branches is significantly large, e.g. the ratio between STT values is ${T}_{||}(V=-20{\rm{mV}})/{T}_{||}(V=+20{\rm{mV}})\approx 5$, which grows up to $\approx$6 at $|V| > 250$ mV due to slight nonlinear behavior with voltage for both cases: $T=250$ mK (Fig. 6a) and $T=300$ K (Fig. 6b), respectively. It was also found that absolute STT magnitude rapidly increases with temperature and might relate with a growth of the spin filtering efficiency on NPs. Derived results may potentially reveal an additional hidden origins of the spin dynamics in NP-MTJs, e.g. utilizing spin-torque magnetic resonance technique of superparamagnetic nanoparticles in MgO-based MTJs by using spin-torque diode effect^29^. Asymmetric STT effect attributes to a valuable benefits for MRAM, which can be potentially fabricated as a system of uniform DMTJ or NP-MTJ rows. We suggest to use this asymmetry to write a bit (initiate magnetization state switching) by negative voltage pulse, since STT is large, while it is more convenient to read a bit in positive voltage range, when STT is much lower.

## Conclusion

In this report, the low temperature anomalies of TMR effects at low voltages were studied in terms of different size distributions of NPs in NP-MTJs. The problem was considered with the dimensional threshold of conducting properties in these systems. Approach of the high degree (or QW) quantization shows precise data fitting at ${d}_{{\rm{av}}} < 1.8$ nm, while the low degree of quantization is more important at ${d}_{{\rm{av}}} > 1.8$ nm, explaining experimental results. In terms of TMR efficiency, the case of uniform middle layer with maximal thickness, allowed for only ballistic tunneling, provides the largest TMR and lowest RA values in comparison to any other size distributions or consequent tunneling cases. For the first time, it was demonstrated that TMR dependencies and in-plane STT components may have quantized step-like voltage behaviors at low temperatures. Finally, calculated NP-MTJ asymmetry and temperature impacts for TMR-*V* curves are in accordance with related experimental observations. Moreover, it was found that barrier asymmetry is very promising for applications, since STT becomes very sensitive for the voltage polarity.

## Electronic supplementary material

ARGUMENTS SUPPORTING THE DOMINANT DIRECT DOUBLE BARRIER TUNNELING
