# Publisher Correction: Symmetric and Asymmetric Magnetic Tunnel Junctions with Embedded Nanoparticles: Effects of Size Distribution and Temperature on Tunneling Magnetoresistance and Spin Transfer Torque

**DOI:** 10.1038/s41598-018-31651-8

**Published:** 2018-10-31

**Authors:** Arthur Useinov, Hsiu-Hau Lin, Chih-Huang Lai

**Affiliations:** 1 Department of Physics, National Tsing Hua University, Hsinchu, Taiwan; 2 Department of Materials Science and Engineering, National Tsing Hua University, Hsinchu, Taiwan; 3 Institute of Physics, Kazan Federal University, Kazan, Russian Federation

Correction to: *Scientific Reports* 10.1038/s41598-017-08354-7, published online 21 August 2017

This Article contains typographical errors.

In the Introduction under subheading ‘Theoretical model’,

“Ten fractions *f* = 1.10 were considered with diameter *d*^(*f*)^, and ∑*fif* = 100, where *i* is a number of NPs.”

should read:

“Ten fractions *f* = 1–10 were considered with diameter *d*^(*f*)^, and ∑*fif* = 100, where *i* is a number of NPs.”

“Parabolic quantization rule was applied in another subsections B to F for low and high temperatures.”

should read:

“Parabolic quantization rule was applied in another subsections for low and high temperatures.”

In the Results under subheading ‘Approach of quantization over QW states’,

“Its NP size distribution, Fig. 2e, demonstrates how NPs numbers ${i}_{f=}\,$2 and ${i}_{f=7}$ is changed in opposite way”

should read:

“Its NP size distribution, Fig. 2e, demonstrates how NPs numbers ${i}_{f=2}$ and ${i}_{f=7}$ is changed in opposite way”

In the Results under subheading ‘Approach of asymmetric NP-MTJ’,

“The problem R → 0 in asymmetric NP-MTJ is considered insupplementary material with presence of the additional intrinsic voltage bias on the interfacesThe negative and positive TMR-V curves reproduce TMR asymmetric behaviors which are highlighted for experimental data in Fig. 4c at t ≈ 0.5 nm (region I) and t ≈ 1.6 nm (region III), respectively.”

should read:

“The problem R (V → 0) in asymmetric NP-MTJ is considered in supplementary material with presence of the additional intrinsic voltage bias on the interfaces. The negative and positive TMR-V curves reproduce TMR asymmetric behaviors which are highlighted for experimental data in Fig. 4c at t ≈ 0.5 nm (region I) and t ≈ 1.6 nm (region III), respectively.”

Additionally, this Article contains a typographical error in the PDF version of this Article. In the Results under subheading ‘Approach of quantization over QW states’,

“The lowest ${k}_{n}^{(f)}$ values corresponds to NPs distribution with greatest averaged NP diameter: ${d}_{av}=1{N}_{{\rm{tot}}}$∑${i}_{f}{d}^{(f)}$.”

should read:

“The lowest ${k}_{n}^{(f)}$ values corresponds to NPs distribution with greatest averaged NP diameter: ${d}_{av}=\frac{1}{{N}_{{\rm{tot}}}}$∑${i}_{f}{d}^{(f)}$.”

