# Supplementary material for: Symmetric and Asymmetric Magnetic Tunnel Junctions with Embedded Nanoparticles: Effects of Size Distribution and Temperature on Tunneling Magnetoresistance and Spin Transfer Torque
Source: Sci Rep. 2017 Aug 21;7:8357. doi: 10.1038/s41598-017-08354-7 (PMC5566438; doi:10.1038/s41598-017-08354-7)
Supplement: Supplementary file 1 — ARGUMENTS SUPPORTING THE DOMINANT DIRECT DOUBLE BARRIER TUNNELING [file 41598_2017_8354_MOESM1_ESM.doc]

Symmetric and asymmetric magnetic tunnel junctions with embedded nanoparticles: Effects of size distribution and temperature on tunneling magnetoresistance and spin transfer torque

Arthur Useinov^1,2,3^, Hsiu-Hau Lin^1^ and Chih-Huang Lai^2^

arguments supporting the dominant direct double barrier tunneling

The first principal estimations presented in Wang’s work [1] show the importance of the quantum well (QW) resonant states in the double barrier system, the middle layer was simulated as continuous Fe thin film. The resonant states are possible to observe for the voltage values which are comparable with Coulomb blockade (CB) energy. The Coulomb charging energy ($V_{\text{CB}}$) via diameter of the metallic NP (*d*) is shown in [Fig. 3]. The picture satisfies to the simple electrodynamic model where $V_{\text{CB}}\propto1/A\propto1/d^{2}$, *A* is area of the NP surface, $V_{\text{CB}}=2e/C$, and capacitance $C=\varepsilon\varepsilon_{0}\pi d^{2}/4d_{\text{MgO}}$. It is assumed that for the small NP the $V_{\text{CB}}$ rapidly increases. Our point is that this suggestion is valid for the case of one or a few NPs in range of the one device, $C$ is small enough to induce the CB effects. The size of the nanoparticles (NPs) is also important because in case of ultra-small NPs the quantum principle of the uncertainty reduce the life-time of the conduction electron on the NP, satisfying the physical limit of the single barrier when thickness of the middle conducting layer is vanished. In the case which is considered in our work the problem have to account large number of NPs, the amount of NPs per unit area increases significantly in case of the small NP *d*< 3.5 nm (this condition shifts the physical treatment of the results derived in Wang’s work in relation to our problem). In such system the parallel connection of the small capacitors (related with NPs) have to be considered as a summation of their capacitance $C_{eff}\approx\sum_{i} C_{i}\approx\varepsilon_{0}\varepsilon_{MgO}/d_{MgO}\sum_{i} A_{i}$ that finally may give the opposite effect for the $V_{\text{CB}}.$ Moreover, the capacitance definition derived for the plain capacitor in classical electrodynamics is based on the assumption of the equipotential surface which can be braked in nano scale: e.g. the Sharvin conductance [2] is result of the increased gradient of the potential due to constricted geometry of the point-like contact, otherwise classical Ohm law is valid. The uncertainty principle $\Delta n\times\Delta\Phi$~ determines the criteria of the scales, $\Delta n$~$\Delta E$ is the number of discrete states in the systems showing the degree of quantization; $\Delta\Phi$~$\Delta z$ is the spectra of interface phases, which responsible for localization ($\Delta z$) of the electron. In case of CB $\Delta n$ is small and $\Delta\Phi$~ have to be large that support the enhanced electron life-time $\tau$ inside NP, the tunneling rate $\Gamma\propto1/\tau$is small. CB effect is important but only when NP collected some critical size $d$ $\approx$ $10 \text{nm}$. In opposite case, when $\Delta n$ is large, *n* becomes not-well defined quantum number and hence CB is absent, the increased tunneling rate $\Gamma$ supports the ballistic transport regime: the ratio between *d* and mean free path of the electron *l* becomes important. It also means that $V_{\text{CB}}$ have to drops down at critically small island size somewhere in the range of *d* = 0.5 $-$ 4.5 nm, however this range of the dimensions is not shown in [Fig.3], Ref.[1].

Furthermore, considering the data [Fig. 2(a) and Fig. 2(b)] of the Wang’s work, the lowest-bias QW resonances at resonant bias $V_{\text{R}}=V_{\text{CB}}$ are shifted to the range of large biases for the small NPs, e.g. $V_{\text{R}}\approx0.2-0.3$ V for *t* = 1.15 nm, while $V_{\text{R}}\approx0.025$V for *t* = 4.74 nm. It means that presence of QW resonances due to CB do not explain low bias ($V_{\text{R}}$~$1.0-3.0 \text{mV}$) TMR anomalies for small particles (*t <* 0.4 nm), which are observed in experimental data: [Fig. 4, Fig. S6] in Ref.[3] for *t* = 0.3 nm, *t* = 1.1 nm, and [Fig. 4] in Ref.[4]. In addition, in our point of view, the reason of the *dV*/*dI* oscillations, which are observed even at room temperature in fully epitaxial double barrier MTJ, Fe/MgO/Fe ( *t* = 1.2 nm, *d* $\approx$ 5 nm) /MgO/Fe in Ref.[5], probably related to the electron-phonon interactions and barrier defects [6] rather than to CB and QW resonance effects which must be totally suppressed at high temperatures due to band broadening. Unfortunately, in range of our model the phonon assisted tunneling, barrier defects and phonon spectra are not considered.

To support the assumption of the bulk-like states in the small metallic NPs the calculations from the first principles were done in range of SIESTA 3.1 at low temperature. We find that the number of states or density of states (DOS) is lower in Fe nanoparticle for *s*- and *p*-type electrons, while it is larger for *d*-type, Fig.1S. The results presented below are shown for cubic and near spherical-like NP (*d* =1.14 nm). In case of *d*-type states, the DOS is large enough to consider the total band structure and dispersion relation as quasi-discontinuous. Thus, small NPs (1.1 nm < *d <* 3.5 nm) have more bulk-like states rather than peak-like or quantum well states. However, it is possible that only *s*- and *p*-type, existing in parallel with *d-*type electrons, can be responsible for the QW resonant states. Finally, in our case, the valuable DOS(*E* =$E_{\text{F}}$) $k_{n}$ allows to apply approach Eq. (2) mainly for *d*-type electrons, which regulate $k_{n}$ as a function of voltage as well as parabolic quantization rule at small applied voltage, showing how fast $k_{n}$ and $E_{\text{F}}$ of the NP increases up with *d* to the bulk level.

| 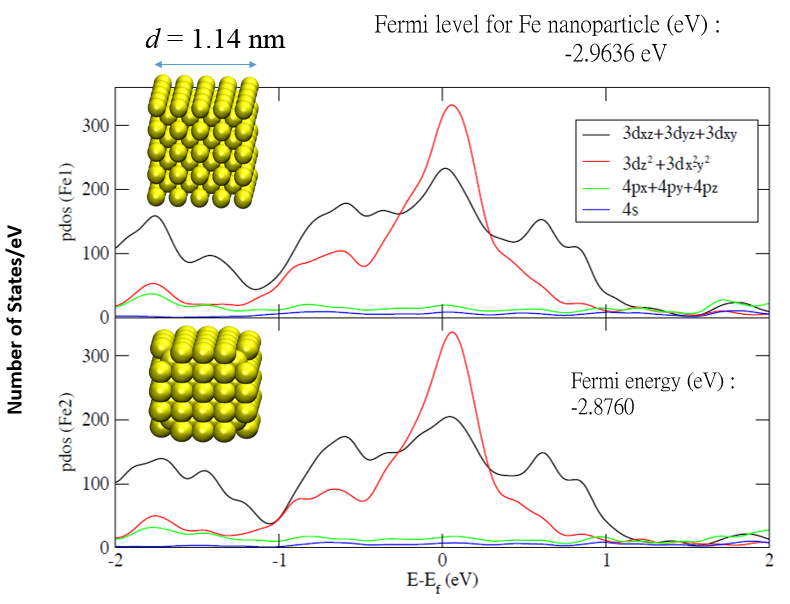 |
| --- |
| Figure 1S: The number of states derived for the iron NP (*d* = 1.14 nm) in range of computational program SIESTA 3.1. The results are close to each other in case of the slight shape modification. |

Furthermore, we are not a first who consider the direct tunneling as a part of the problem. According the tunneling model shown by Ciudad *et al.* Ref.[4] in [section III] there is a competition between direct tunneling and consecutive terms of the conductance, however the critical size for it was not estimated. The tunneling conductance was represented as the summation of the three terms: $\sigma=a \sigma_{D}+b\left(\sigma_{E}+\sigma_{K}\right)$, where $a$ + *b* = 1, $\sigma_{K}$ is the conductance through the NPs due to Kondo effect; $\sigma_{D}$ and $\sigma_{E}$ are the direct tunneling and elastic conductance through the NPs without spin flips, respectively. The contribution for $a$ and *b* depends on the fractional populations of clusters and temperature. Conditions for $a$ and *b* are related to barrier thickness: it assumes $a$ > *b* for the thin layers. The critical thickness for $a$ > *b* is not defined, but assumed that $t\to0$, $a\to1$, while for $t\approx3 \text{nm}$,$a\to0$ and thus $b\to1$, and, in turn, the second term becomes dominant. It was also suggested that $\sigma_{K}$>> $\sigma_{E}$ for$t\approx3 \text{nm}$, but for further *t* increasing it results to $\sigma_{K}\approx\sigma_{E}$ and $\sigma_{E}$ >> $\sigma_{K}$. The competition between $\sigma_{K}$ and $\sigma_{E}$ reflects TMR dependence and its amplitude changes from reduced to enhanced one at small temperatures and voltages. However, nevertheless on the suggested approach and valid estimations for *t* ~3 nm and *t* >3 nm, Ciudad considered the samples *t* < 1.2 nm yet assuming$a\to0$. In turn, the cluster sizes, which are shown in Fig. 1(b) Ref.[4], have roughly *d*_av_ ~ 4.0 - 4.5 nm at *t* = 1.1 nm, and the number of clusters with *d =*10 nm is negligibly small. It should be noticed, the NP size distribution for *t* = 3 nm have to be shifted into the much larger *d*_av_. Moreover, there is no critical size estimations to assume $b\to1$ for the considered cases at *t* < 1.2 nm. According our fist principal calculations shown above: the DOS for the small NP is large enough to be over the CB effects. It is worth to notice also, that formation of the homogeneous layer due to deposition or epitaxial growth of the middle layer may be realized at *t* ~ 1.6 – 2.0 nm, avoiding the case of the dominated and separated clusters with *d*_av_ $\approx$ 10 nm (*t* $\approx$ 3 nm), where CB might be important.

Mean free paths (*l*) is an important parameter showing the scale in the system and determining the conditions for the ballistic (direct) transport. For example, *l* = 5.5 nm in the Co thin films for majority electrons at room temperature [7]. The mean free path in the bulk is larger, e.g. depending from transport direction perpendicular to hexagonal (tetragonal) axis *l* = 11.8 nm (7.77 nm) for the crystal Co, respectively [8]. These values are comparable or larger with the maximal total tunneling thickness of complex barrier in our system. The *l* increases at least in a few times at low temperatures and, thus *l* becomes more than 20.0 nm that is much larger than the total barrier thickness even including the cases *d* > 3.5 nm.

Assuming that the barrier (or both barriers) in single or in double barrier system are vanished, the case of point-like contact with radius $a_{0}$ occurs. The conditions $a_{0}$*<< l* or $a_{0}$ $\approx$ *l*  may happened when metallic NP touches the both ferromagnetic layers. And all transport properties should reflect the physics of point-like contact or point-contacts connected in parallel, and, thus, the transport model have to be conjugated with Sharvin limit. In turn, our approach validity for ballistic and diffusive transport in point-like contact is shown in supplementary material of the previous work [9] as well as in Ref.[10].

As a result, we claim that direct tunneling approach (*d* < 3.5 nm) with ballistic double barrier scattering potential explains TMR anomalies and TMR asymmetry (Fig. 2b, Fig. 5a and Fig. 5b) much better than the model of CB with related consecutive models which are suggested initially by Wang *et al.* [1], Yang *et al.* [3] and Ciudad *et al.* [4]. However, we agreed that the CB as well as other related effects can be dominant over direct tunneling at low concentration of NP inside the barrier for *d* >> 3.5 nm, but this case is out of our consideration.

problem of the resistance behavior in asymmetric np-mtj at zero voltage

The model is also convenient for *I-V* and *R-V* simulations in NP-MTJs with barrier-related asymmetry with presence of the intrinsic voltage bias $V_{01\left(02\right)}$. The intrinsic voltage arises due to the contact potential difference between interfaces that shifts TMR peak or TMR dip into the finite positive or negative voltage range (e.g. exp. data in Fig.2b). The main results of the letter are obtained for $V_{01\left(02\right)}\to0$. In the case of valuable intrinsic bias $V_{1(2)}=V\cdot\varepsilon_{2(1)}L_{1(2)}/\left(\varepsilon_{2}L_{1}+\varepsilon_{1}L_{2}\right)\pm V_{01(02)}$, and in case of the voltage reversal $V_{1(2)}\to V_{2(1)}$, while $\pm V_{01(02)}\to\mp V_{02(01)}$. The asymmetric voltage drop is proportional to the barrier width resulting in semiconductor-free diode effect [11,12]

| 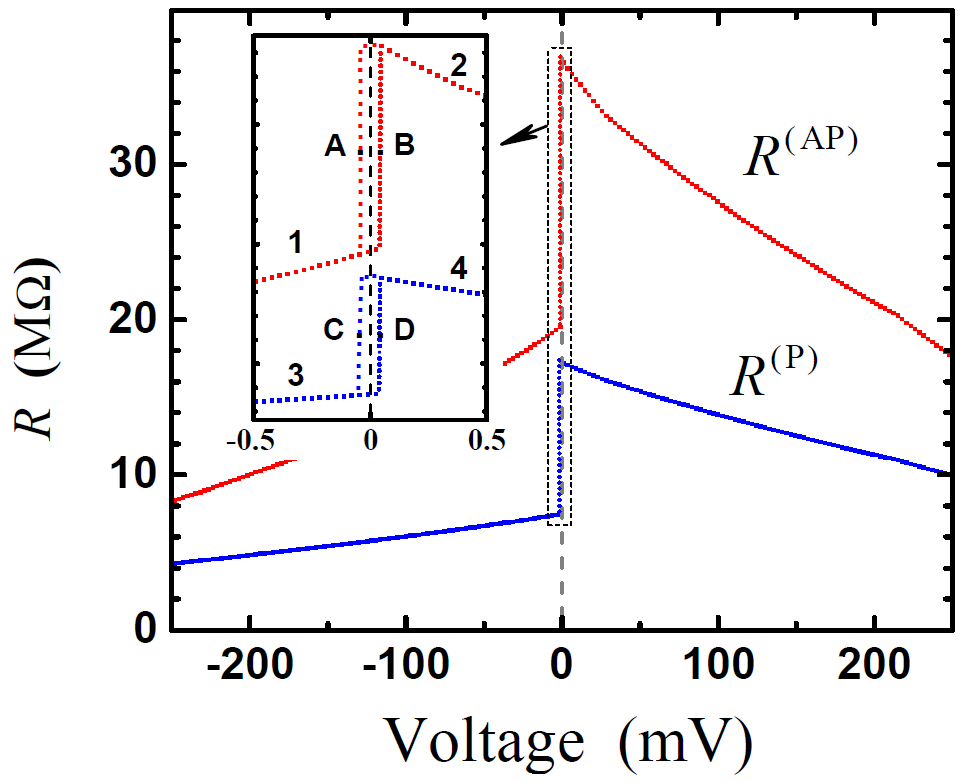 |
| --- |
| Figure 2S: Asymmetric resistance behavior $L_{1}=0.9$nm, $L_{2}=1.0$nm for $d_{\text{av}}=2.63$nm. The inset show the intrinsic bias range, where $\left\vert V_{\text{A}}-V_{\text{B}}\right\vert=\left\vert V_{\text{C}}-V_{\text{D}}\right\vert=2\left\vert V_{01}+V_{02}\right\vert\approx0.1$mV. |

The *R-V* curves are shown as example in Fig. 2S with small intrinsic voltage. The curves behave in two different ways depending from sign of the intrinsic bias as it shown in the inset. The paths 1A2, 3C4 and 1B2, 3D4 correspond to $+V_{01\left(02\right)}$ and $-V_{01\left(02\right)}$, respectively. The presence of $V_{01\left(02\right)}$ solves the problem $R(-0)\approx R(+0)$ at $V\to0$. The asymmetry can be also induced by different top and bottom FML and related $k_{F,s}$, but this kind of asymmetry is out of our consideration.

# Acknowledgment

We are thankful to Prof. Chao Cheng Kaun (Academia SINICA of Taiwan) and Dr. Ching-Hao Chang (Department of Physics, NTHU) for the derived results shown in Fig.1S.

References

1. Wang, Y. *et al.* First-principles theory of quantum well resonance in double barrier magnetic tunnel junctions. *Phys. Rev. Lett.* **97,** 087210 (2006)
2. Sharvin*,* Yu. V. A possible method for studying Fermi surfaces. *Sov. Phys. JETP* **21,** 655 (1965)
3. Yang, H. *et al.* Crossover from Kondo-assisted suppression to co-tunneling enhancement of tunneling magnetoresistance via ferromagnetic nanodots in MgO tunnel barriers. *Nano Lett.* **8,** 340-344 (2008)
4. Ciudad, D. *et al.* Competition between co-tunneling, Kondo effect, and direct tunneling in discontinuous high-anisotropy magnetic tunnel junctions. *Phys. Rev. B.* **85,** 214408 (2012)
5. Nozaki, T. *et al.* Quantum oscillation of the tunneling conductance in fully epitaxial double barrier magnetic tunnel junctions. *Phys. Rev. Lett.* **96,** 027208 (2006)
6. Naidyuk, Yu. G.; Yanson, I.K. *Point-Contact Spectroscopy* (Copyright 2005 Springer Science & Business Media Inc.), p.9, p.38.
7. Gurney, B.A. *et al.* Direct measurement of spin-dependent conduction-electron mean free paths in ferromagnetic metals. *Phys. Rev. Lett.* **71,** 4023 (1993)
8. Gall, D. Electron mean free path in elemental metals. *J. Appl. Phys.* **119,** 085101 (2016)
9. Useinov, A. *et al.* Anomalous tunnel magnetoresistance and spin transfer torque in magnetic tunnel junctions with embedded nanoparticles. *Sci. Rep.* **5,** 18026 (2015)
10. Useinov, A. *et al.* Tunnel magnetoresistance and temperature related effects in magnetic tunnel junctions with embedded nanoparticles. *SPIN* **6,** 1650001 (2016)
11. Kaniouri, F., *et al.* Diode effect in magnetic tunnel junctions with impurities. *J. Appl. Phys.* **98,** 083901 (2005)
12. Iovan, A., *et al.* Spin Diode Based on Fe/MgO Double Tunnel Junction. *Nano Lett.***8,** 805 (2008).
